# SMAR1 coordinates HDAC6-induced deacetylation of Ku70 and dictates cell fate upon irradiation

**DOI:** 10.1038/cddis.2014.397

**Published:** 2014-10-09

**Authors:** N Chaudhary, K K Nakka, P L Chavali, J Bhat, S Chatterjee, S Chattopadhyay

**Affiliations:** 1 Chromatin and Disease Biology Laboratory, National Centre for Cell Science, Pune University Campus, Pune, India; 2 Department of Biophysics, Bose Institute, Kolkata, India

## Abstract

Acetylation status of DNA end joining protein Ku70 dictates its function in DNA repair and Bax-mediated apoptosis. Despite the knowledge of HDACs and HATs that are reported to modulate the acetylation dynamics of Ku70, very little is known about proteins that critically coordinate these key modifications. Here, we demonstrate that nuclear matrix-associated protein scaffold/matrix-associated region-binding protein 1 (SMAR1) is a novel interacting partner of Ku70 and coordinates with HDAC6 to maintain Ku70 in a deacetylated state. Our studies revealed that knockdown of SMAR1 results in enhanced acetylation of Ku70, which leads to impaired recruitment of Ku70 in the chromatin fractions. Interestingly, ionizing radiation (IR) induces the expression of SMAR1 and its redistribution as distinct nuclear foci upon ATM-mediated phosphorylation at serine 370. Furthermore, SMAR1 regulates IR-induced G2/M cell cycle arrest by facilitating Chk2 phosphorylation. Alternatively, SMAR1 provides radioresistance by modulating the association of deacetylated Ku70 with Bax, abrogating the mitochondrial translocation of Bax. Thus, we provide mechanistic insights of SMAR1-mediated regulation of repair and apoptosis via a complex crosstalk involving Ku70, HDAC6 and Bax.

Nuclear matrix (NM) is a fibrogranular network and an active site for various nuclear events, such as recombination, repair, splicing, transcription and so on.^1^ NM functions as a scaffold for DNA double-strand break (DSB) repair as various repair factors are associated with its filamentous structure upon DNA damage.^2,3^ Matrix attachment region-binding proteins (MARBPs) are unique class of proteins that bind to specific non-coding sequences in the genome termed as scaffold/matrix attachment regions, and globally modify the topology of chromatin.^4^ Scaffold/matrix-associated region-binding protein 1 (SMAR1) is one such MARBP, which was first identified in mouse double positive thymocytes.^5^ SMAR1 exhibits transcriptional repression of multiple genes^6,7^ and responds to various kinds of stress.^8,9^

Ku70, a key player of non-homologous end joining (NHEJ) repair pathway,^10^ associates with NM and acts as a docking factor to promote the tethering of free DSB ends to NM for repair.^3,11, 12, 13^ Posttranslational modification of many repair proteins has a prominent role in controlling the spatiotemporal dynamics of such factors at the site of damaged DNA. For example, modulation of Ku70 acetylation is a key switch between the two contrasting cellular fates upon stress: repair and death.^14, 15, 16^ Ku70 acetylation inversely correlates with its DNA-binding property and repair efficiency.^17^ Deacetylated Ku70 interacts and sequesters cytoplasmic pro-apoptotic protein Bax,^16,18^ but the acetylation of Ku70 at its C-terminus leads to disruption of Ku70–Bax complex and mitochondrial translocation of Bax to induce apoptosis.^14,19^ Positive regulation of cell survival upon stress is mediated through Ku70 deacetylation by various histone deacetylases, such as HDAC6,^17,18,20^ SIRT1,^15^ and SIRT3.^21^ However, underlying mediator/regulatory proteins that modulate the deacetylation of Ku70 in response to stress remain enigmatic.

In the present study, we delineated a complex molecular mechanism of DNA damage repair and cell survival upon ionizing radiation (IR)-induced cellular stress. We found that SMAR1 is a novel interacting partner of Ku70 and mediates HDAC6-induced deacetylation of Ku70. Although it is established by various groups that HDAC6 deacetylates Ku70, we provide substantial evidence to prove the indispensability of SMAR1 for HDAC6-mediated Ku70 deacetylation. Multiple experiments establish that SMAR1, HDAC6 and Ku70 exist in the form of triple complex, with SMAR1 functioning as an intermediate bridge between HDAC6 and Ku70. We also show that upon IR, SMAR1 is phosphorylated at serine 370 by ATM and relocates to DSB sites. Furthermore, overexpression of SMAR1 favors IR-induced G2/M arrest, whereas its knockdown results in inefficient DNA repair and diminished cell survival. SMAR1 displays functional inhibition of Bax by regulating Ku70–Bax association. Together, our study demonstrates the novel role of SMAR1 in coordinating an intricate molecular mechanism upon DNA damage through modulation of Ku70 deacetylation.

## Results

### SMAR1 is induced upon irradiation and interacts with Ku70

**Figure 1:**
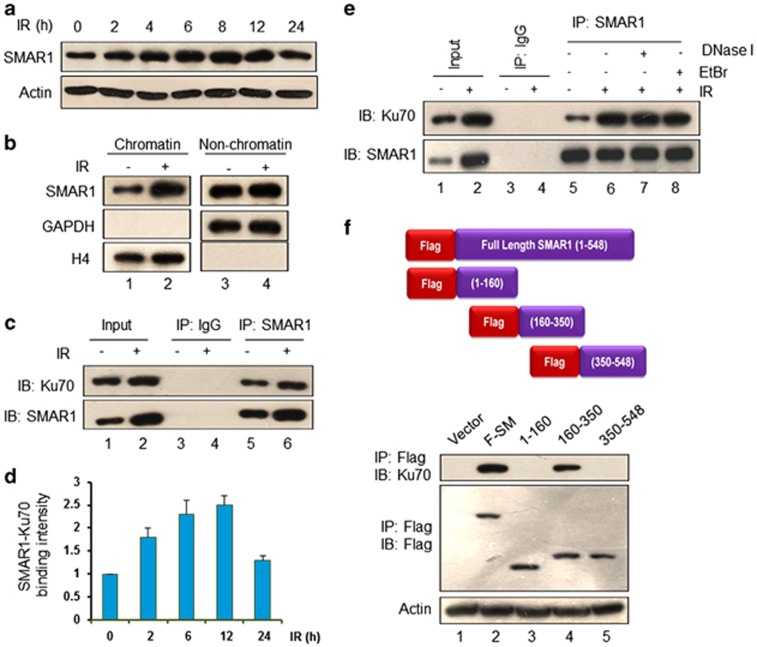
SMAR1 interacts with Ku70. (**a**) HCT116 cells were exposed to ionizing irradiation (10 Gy) followed by immunoblot analysis of SMAR1 at the indicated time points. Actin was used as a loading control. (**b**) Western blot analysis of SMAR1 and mentioned control proteins in chromatin and non-chromatin fractions of HCT116 cells that were either left untreated or irradiated (10 Gy, 2 h). (**c**) IP assay to check the interaction of SMAR1 with Ku70 upon IR (10 Gy, 8 h), wherein cell lysates were first immunoprecipitated with SMAR1 or control immunoglobulin G (IgG) followed by immunoblotting of the eluates with indicated antibodies, as shown. In all, 20% fraction of the whole-cell extract served as input control. (**d**) IP assay to study SMAR1–Ku70 association upon IR (10 Gy) in HCT116 cells at indicated time points. Graph depicts the densitometry quantification of time-dependent interaction between SMAR1 and Ku70 using QuantityOne software (VersaDoc imaging system, BioRad). Control cell sample (as the value of 1) was used to normalize values of treated samples and data represents mean±S.D. from three independent experiments. (**e**) Control and irradiated (10 Gy) HCT116 lysates were treated with either EtBr (50 *μ*g/ml, 30 min) at 4 °C or DNase I (100 U/ml, 20 min) at 37 °C. Whole-cell lysates were immunoprecipitated with SMAR1 and then immunoblotted for Ku70 and SMAR1. (**f**) To study domain-specific interaction between SMAR1 and Ku70, HCT116 cells were transfected with Flag-tagged SMAR1 constructs (upper panel) and lysates were immunoprecipitated with anti-Flag antibody followed by immunoblotting with indicated antibodies (lower panel)

Studies from our laboratory had shown that SMAR1 is a stress-responsive protein, but least is known about its regulatory role during IR-induced DNA damage. Our initial observations in HCT116 cells revealed an induction in the expression of SMAR1 in a dose (Supplementary Figure S1a) and time-dependent manner upon IR (Figure 1a and Supplementary Figure S1b). Considering that the recruitment of certain factors to chromatin-associated DSB sites is a prerequisite for efficient repair,^22^ we investigated the expression levels of SMAR1 in the chromatin and non-chromatin fractions upon irradiation. Results showed a considerable increase in the chromatin-associated SMAR1 upon IR (Figure 1b, lane 2 and Supplementary Figure S1c). Considering that Ku70, a key modulatory protein of NHEJ repair pathway, is recruited to chromatin upon IR,^23^ we investigated its association with SMAR1. Immunoprecipitation (IP) assays in control and irradiated HCT116 cells (10 Gy, 8 h) showed that SMAR1 interacts with Ku70 even in the absence of DNA damage (Figure 1c, lanes 5 and 6, respectively and Supplementary Figure S1d). Despite the discrepancies about Ku70 induction upon IR, some reports suggest increased expression of Ku70.^24^ Similarly, we observed increased Ku70 in irradiated cells (Figure 1c, lane 2). The interaction of SMAR1 with Ku70 was further validated by reverse IP in HCT116 cells, *in vivo* interaction studies in different cell lines, such as HEK 293 and MCF-7 cells, and *in vitro* GST pull-down assays (Supplementary Figures S2a–d).

Further, we looked into the binding kinetics of SMAR1 with Ku70 upon DNA damage. Results showed that SMAR1 and Ku70 interaction gradually increases upon IR, reaching to a maximum extent by 12 h, and again restores to the basal level by 24 h (Figure 1d and Supplementary Figure S2e). Next, we explored the possibility of a DNA-mediated interaction between SMAR1 and Ku70 because both SMAR1 and Ku70 are enriched in chromatin-bound fractions upon IR. Surprisingly, interruption of DNA–protein interactions by using DNase I or ethidium bromide (EtBr) did not perturb the association of SMAR1 with Ku70 (Figure 1e), excluding the possibility of independent binding of SMAR1 and Ku70 to the damaged DNA and suggested the existence of SMAR1–Ku70 complex even in the absence of DNA damage. It is noted that interaction of Ku70 with DNA-dependent protein kinase catalytic subunit (DNA-PKcs) is DNA-dependent,^25^ hence served as a positive control (Supplementary Figure S2f). We further mapped the domain of SMAR1 that associates with Ku70 by using a series of Flag-tagged full-length (F-SM) and deletion constructs of SMAR1. Results showed that SMAR1 associates with Ku70 through its protein-interacting domain (160–350 residues) (Figure 1f, lane 4). Neither the N-terminal region (1–160 residues) nor the DNA-binding domain (350–548 residues) of SMAR1 showed any interaction with Ku70 (Figure 1f, lanes 3 and 5). Altogether, we conclude a specific crosstalk between SMAR1 and Ku70.

### SMAR1 modulates the deacetylation of Ku70 via HDAC6

**Figure 2:**
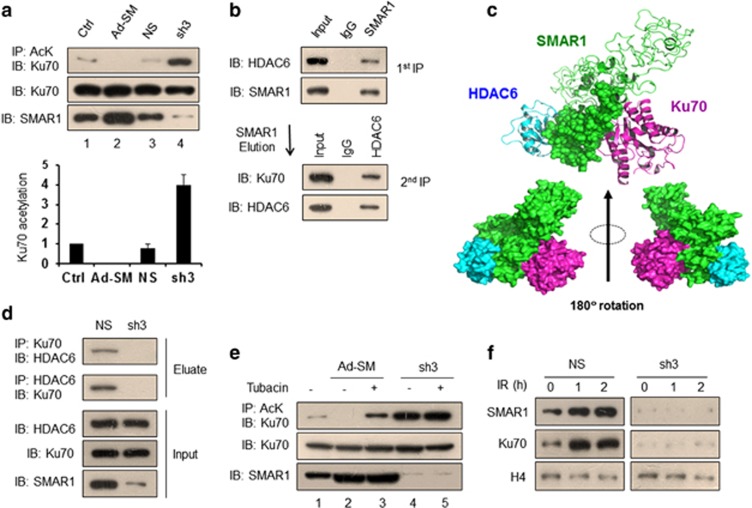
SMAR1 modulates the deacetylation of Ku70 via HDAC6. (**a**) Acetylation-specific IP assay in control cells, cells overexpressed for SMAR1 (Ad-SM), NS and sh3 lentivirus-transduced cells. Cell lysates were immunoprecipitated with anti-acetyllysine (AcK) antibody and immunoblotted with Ku70 antibody. Graph (lower panel) represents the densitometry quantification of mean Ku70 acetylation±S.D. of three independent experiments using QuantityOne software (VersaDoc imaging system, BioRad). (**b**) Sequential IP assay to check the *in vivo* association of SMAR1, Ku70 and HDAC6 in one complex. HCT116 cell lysate (1 mg) was first immunoprecipitated with SMAR1 antibody and eluate was subsequently probed with indicated antibodies, followed by second IP with HDAC6 antibody and immunoblotting with mentioned antibodies. (**c**) IP assay in NS and sh3 lentivirus-transduced HCT116 cells to investigate the association between Ku70 and HDAC6. Whole-cell lysates were immunoprecipitated with either Ku70 or HDAC6, and then immunoblotted with indicated antibodies. (**d**) *In silico* analysis of HDAC6–SMAR1–Ku70 complex. C-terminal of SMAR1 (green spheres) is sandwiched between Ku70 (magenta) and HDAC6 (cyan) (upper panel). Anterior and posterior view of the triple complex (lower panel). (**e**) Protein lysates extracted from control and tubacin-treated (10 *μ*M, 2 h) HCT116 cells, which were previously transduced with either Ad-SM or sh3 lentivirus were analyzed for Ku70 acetylation by IP with anti-AcK antibody and immunoblotting for Ku70. (**f**) Western blot analysis to check IR-induced recruitment of SMAR1 and Ku70 to the chromatin in HCT116 cells that were previously transduced with either NS or sh3 lentivirus

Acetylation of Ku70 has a modulatory role during DNA damage response.^17^ Maintenance of p53 protein in a deacetylated state by SMAR1 through its interaction with HDAC1 ^7^ intrigued us to analyze SMAR1-mediated modulation of Ku70 acetylation dynamics. Towards this, we performed acetylation-specific IP assays in HCT116 cells that were transduced with either SMAR1-adenovirus (Ad-SM) or SMAR1-ShRNA (sh3) lentivirus for the overexpression and knockdown of SMAR1, respectively. Results showed that SMAR1 maintains Ku70 in a deacetylated state, whereas knockdown of SMAR1-induced Ku70 acetylation by approximately fourfold (Figure 2a, right panel). Considering that SMAR1 does not harbor intrinsic deacetylase activity, we investigated the underlying mechanism of SMAR1-mediated Ku70 deacetylation. It is noted that HDAC6 deacetylates Ku70,^18^ hence we explored for a possible co-existence of SMAR1, Ku70 and HDAC6 in one complex. Toward this, sequential IP assays in control HCT116 cells was performed, wherein whole-cell extracts were first immunoprecipitated with SMAR1 and the eluates were further pulled with HDAC6 and consequently probed for the presence of Ku70. Results showed an endogenous association of SMAR1, HDAC6 and Ku70 in a complex (Figure 2b).

We further determined the association pattern of HDAC6–SMAR1–Ku70 complex at the molecular level by performing protein–protein docking analysis. The energy calculations of SMAR1, SMAR1–Ku70, SMAR1–HDAC6 and SMAR1–Ku70–HDAC6 complexes were carried out by using MacroModel module of Maestro (Schrodinger, New York, NY, USA). From the energy content analysis, we observed that the electrostatic interactions due to formation of salt bridges between positively and negatively charged residues in the heterodimers and trimers predominates over all other van der Waals and solvent–solute interactions (Supplementary Table S1). The order of stability (highest to lowest) in different complexes have been found as (SMAR1+Ku70+HDAC6)_Trimer_>(SMAR1+Ku70)_Dimer_>(SMAR1+ HDAC6)_Dimer_>SMAR1_Monomer_. The trimeric model of SMAR1 bound to HDAC6 and Ku70 revealed that 240–350 residues of SMAR1 interact with the N-terminal region of Ku70 through various inter residual salt bridge formation. *In silico* analysis of HDAC6–SMAR1–Ku70 docked model revealed that Ku70 is bound to SMAR1 adjacent to HDAC6-binding site (Figure 2c). It was observed that C-terminal domain (248–371 residues) of SMAR1 is sandwiched between Ku70 and HDAC6. To further substantiate SMAR1-mediated interaction of Ku70 with HDAC6, we checked the Ku70–HDAC6 association upon SMAR1 knockdown. Results showed that the specific interaction of HDAC6 with Ku70 was retained in cells transduced with non-silencing (NS) lentivirus; however, the knockdown of SMAR1 with sh3 lentivirus perturbed the interaction (Figure 2d).

Altogether, these data suggest that SMAR1 has a critical role in HDAC6-mediated deacetylation of Ku70. In order to further validate SMAR1-mediated Ku70 deacetylation via HDAC6, we investigated the effect of HDAC6-specific inhibitor tubacin on the Ku70 deacetylation. IP assays revealed that tubacin partially hampered SMAR1-mediated Ku70 deacetylation, suggesting that SMAR1 coordinates Ku70 deacetylation via HDAC6 (Figure 2e, lane 3). As deacetylated form of Ku70 is reported to be efficiently recruited to the sites of damaged DNA, we then checked Ku70 enrichment in the chromatin fractions of NS and sh3 lentivirus-transduced HCT116 cells upon IR. The recruitment of Ku70 to chromatin was convincingly impeded in the absence of SMAR1 (Figure 2f, right panel). Furthermore, recruitment of Ku70 to laser microirradiation-induced DNA damage sites was severely impaired upon siRNA-mediated ablation of SMAR1 (Supplementary Figures S3a and b), which further affirms that SMAR1 affects the ability of Ku70 to bind DSB sites. Together, these results suggest that SMAR1 functions as a mediator for HDAC6-induced deacetylation of Ku70 and thus facilitates its chromatin recruitment.

### ATM is indispensable for SMAR1 function upon DNA damage

**Figure 3:**
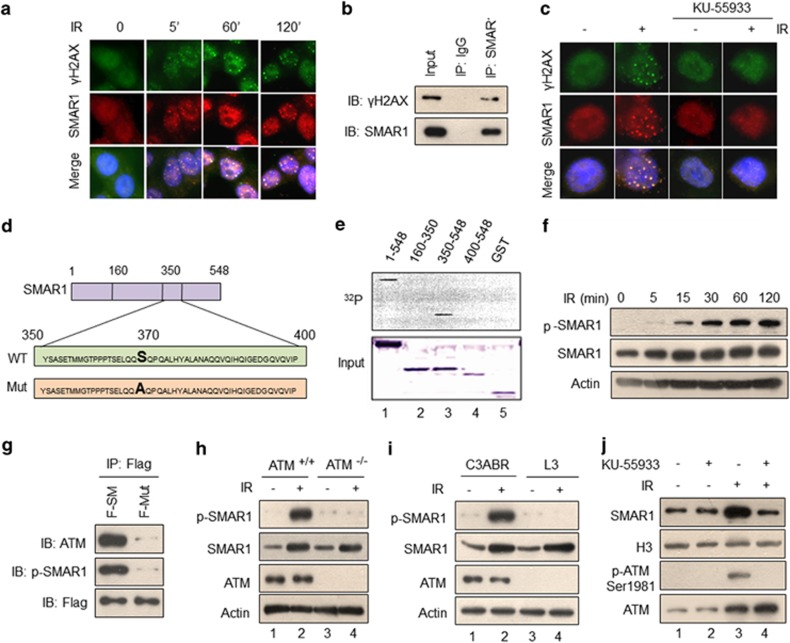
SMAR1 interacts with *γ*H2AX and is phosphorylated by ATM at serine 370 upon IR. (**a**) Immunofluorescence analysis of HCT116 cells for SMAR1 (red) and *γ*H2AX (green) upon IR (10 Gy) at indicated time points. DAPI (blue) was used to stain nucleus. (**b**) IP assay to check interaction between *γ*H2AX and SMAR1 upon IR (10 Gy, 2 h). Irradiated HCT116 cell lysates were immunoprecipitated with SMAR1 and the eluates were immunoblotted with the indicated antibodies. (**c**) Immunofluorescence analysis of SMAR1 (red) and *γ*H2AX (green) in HCT116 cells that were either left untreated or pretreated with KU-55933 (10 *μ*M, 2 h) before IR (10 Gy). (**d**) A Schematic representation of SMAR1 protein sequence showing different regions along with a potential ATM phosphorylation site serine 370 in wild-type SMAR1(WT), which is substituted by alanine in mutant form of SMAR1 (Mut). (**e**) ATM-mediated phosphorylation of SMAR1 was studied by performing *in vitro* kinase assay. Immunoprecipitated ATM from irradiated cell lysate was incubated with 1 *μ*g each of recombinant GST-SMAR1 (1–548), various GST-tagged truncations of SMAR1 and GST alone. Phosphorylation of SMAR1 was observed by autoradiography. The panel below depicts coomassie staining of all recombinant proteins used for kinase assays. (**f**) Immunoblot analysis of SMAR1 phosphorylation in HCT116 cells upon irradiation (10 Gy) using anti-phospho-SMAR1 antibody at indicated time points. (**g**) IP assay to study the association of SMAR1 with ATM in irradiated HCT116 cells (10 Gy, 2 h) transfected with either Flag-SMAR1 (F-SM) or Flag-mutant (F-Mut). Whole-cell lysates were immunoprecipitated with anti-Flag antibody followed by immunoblotting with indicated antibodies. (**h** and **i**) Immunoblotting of phospho-SMAR1 and indicated proteins in MEFs from ATM^+/+^ and ATM^−/−^ mice (**h**), and A-T cell lines such as C3ABR and L3 (**i**) upon IR (10 Gy, 2 h). (**j**) Western blot analysis of SMAR1 and mentioned proteins in chromatin fractions from HCT116 cells that were treated with KU-55933 (10 *μ*M, 2 h) before irradiation

The proteins such as 53BP1, Mre11, BRCA1 and so on are recruited to the sites of DNA damage and form IR-induced foci.^26, 27, 28^ Considering the enrichment of SMAR1 in the chromatin fraction upon IR, we checked for IR-induced relocalization of SMAR1 in the nucleus. Immunofluorescence analysis of SMAR1 in HCT116 cells revealed that it forms distinct nuclear foci as early as 5 min post IR (Figure 3a). Interestingly, these foci were found to colocalize with IR-induced *γ*H2AX foci. To further corroborate the recruitment of SMAR1 to DSB sites, we employed laser microirradiation approach to introduce DNA damage in a distinct tract (laser line) inside the cell nucleus. Understandably, we observed enrichment of SMAR1 as well as its colocalization with *γ*H2AX along the laser-induced damaged DNA tracts (Supplementary Figure S4a). Furthermore, IP assays in irradiated HCT116 cells indicated the association of SMAR1 with *γ*H2AX (Figure 3b and Supplementary Figure S4b), suggesting that SMAR1 might function as an early-response protein upon DNA damage. Furthermore, we investigated whether ATM kinase affects the redistribution of SMAR1 to such foci. To test this, HCT116 cells were treated with ATM kinase inhibitor KU-55933 (10 *μ*M, 2 h) before irradiation. Results showed abrogation of IR-induced SMAR1 foci formation upon ATM inhibition (Figure 3c). Taken together, these data indicate that SMAR1 relocalizes inside the nucleus to form foci in response to DNA damage in an ATM kinase-dependent manner.

Next, we examined whether SMAR1 is a substrate of ATM kinase. Initially, scanning of SMAR1 amino-acid sequence revealed the presence of a consensus ATM phosphorylation site SQ (a serine followed by glutamine; Figure 3d).^29^ To establish ATM-mediated phosphorylation of SMAR1, *in vitro* kinase assays were performed with recombinant full-length GST-SMAR1 and various truncations, such as GST-SMAR1 (160–350 residues), GST-SMAR1 (350–548 residues) and GST-SMAR1 (400–548 residues). Results showed the phosphorylation of full-length SMAR1 as well as GST-SMAR1 (350–548 residues), but not GST-SMAR1 (160–350 residues) and GST-SMAR1 (400–548 residues) (Figure 3e, lanes 1 and 3), which can further be explained owing to the presence of a single SQ motif in the 350–400 region of SMAR1 (see also Figure 3d). To further confirm IR-induced ATM-mediated phosphorylation of SMAR1, we generated anti-phosho-SMAR1 antibody that specifically recognizes serine 370 phosphorylated form of SMAR1 (p-SMAR1). Time-course study for SMAR1 phosphorylation in irradiated HCT116 cells using p-SMAR1 antibody indicated that phosphorylation of SMAR1 begins as early as 5 min upon IR (Figure 3f, lane 2). IR dose, as low as 0.5 Gy, induced significant phosphorylation of SMAR1, although we observed a proportionate increase in p-SMAR1 with the higher doses of IR (Supplementary Figure S4c, lanes 2 and 7). The specificity of phospho-antibody was further illustrated by probing the Flag-immunoprecipitated eluates from control and irradiated HCT116 cells that were exogenously expressed with either F-SM or phosphorylation-deficient S370A SMAR1 mutant (F-Mut, substituting serine with alanine). The presence of a specific band in irradiated F-SM-transfected cells, but failure of phospho-SMAR1 antibody to recognize mutant SMAR1 (Supplementary Figure S4d, lanes 2 and 4), highlighted the specificity of the antibody and established that serine 370 is a potential site for IR-induced phosphorylation of SMAR1 by ATM kinase. The association of ATM with wild-type SMAR1, but not with phosphorylation-deficient (S370A) SMAR1 in irradiated HCT116 cells (Figure 3g), suggested that ATM directly associates with SMAR1 by recognizing serine 370 as its consensus phosphorylation site and mutation of this site abrogates the ATM association with SMAR1, hence mutant SMAR1 remains unphosphorylated.

The irradiated ATM^+/+^ mouse embryonic fibroblasts (MEFs) and human A-T lymphoblastoid control C3ABR cells showed phosphorylation of SMAR1, whereas ATM^−/−^ MEFs and ATM-deficient lymphoblastoid L3 cells exhibited complete abrogation of SMAR1 phosphorylation (Figures 3h and i). As ATM-mediated DNA damage signaling is considered to be indispensable for efficient recruitment of repair proteins to DSB sites,^30^ we wondered whether the redistribution of SMAR1 to damage-associated chromatin fraction is ATM-dependent. Results showed that treatment with ATM inhibitor KU-55933 (Figure 3j) and PI3 kinase inhibitor caffeine (Supplementary Figure S4e), before irradiation, substantially reduced the chromatin enrichment of SMAR1. Altogether, results from these experiments strongly indicated that ATM-mediated DNA damage signaling is essential for the recruitment of SMAR1 to the site of damaged DNA.

### SMAR1 is essential for DNA repair and favors G2/M arrest

**Figure 4:**
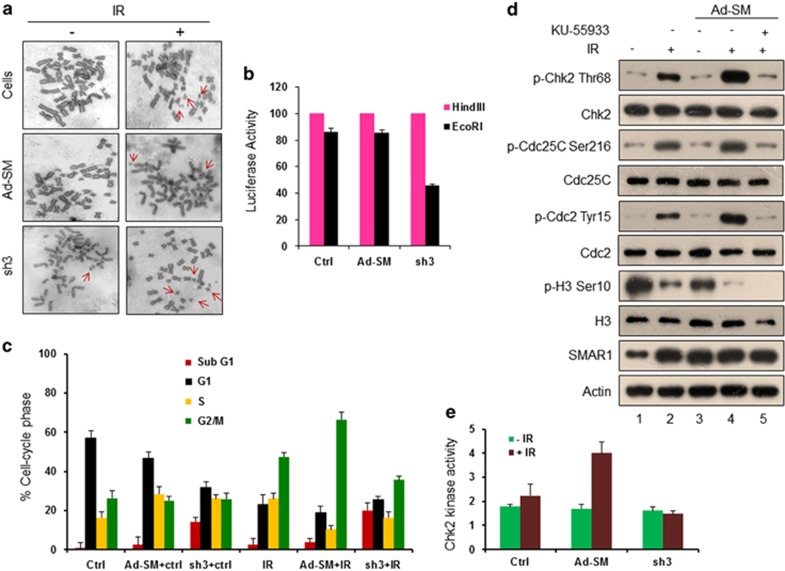
SMAR1 favors G2/M cell cycle arrest through Chk2 phosphorylation. (**a**) Metaphase spread analysis of SMAR1 overexpressed (Ad-SM) and knockdown (sh3) HCT116 cells upon IR (10 Gy). Red arrows indicate chromosomal aberrations. The data are representative of >50 images (*n*>50), which were acquired in various fields from three independent experiments. (**b**) *In vivo* NHEJ assay upon SMAR1 overexpression (Ad-SM) and knockdown (sh3) in HCT116 cells cotransfected with a linearized pGL2-Luc plasmid that was previously digested with either HindIII (purple bar) or EcoRI (black bar), and pRL-SV40 renilla luciferase vector. The luciferase enzyme activity was normalized by dividing the Luc signal with the renilla signal. Error bars represent S.D. from three independent experimental repeats. (**c**) PI staining to analyze the effect of SMAR1 on IR-induced cell cycle progression. Statistical representation of different cell cycle phases upon irradiation (10 Gy, 48 h) in control, and SMAR1 overexpressed (Ad-SM) or knockdown (sh3) HCT116 cells. Error bars represent S.D. from three independent experiments. (**d**) Western blot analysis of indicated proteins in control and SMAR1 overexpressed (Ad-SM) HCT116 cells that were treated either with or without IR (10 Gy, 4 h) and KU-55933 (10 *μ*M, 2 h). (**e**) Chk2 kinase activity assay in the presence and absence of SMAR1 in control (green bar) and irradiated (brown bar) HCT116 cells (10 Gy, 4 h). Error bars represent S.D. from three independent experimental repeats

To further investigate the functional significance of SMAR1 in DSB repair and characterize the cytological consequences of SMAR1 deficiency in response to DNA damage, we examined the chromosome spreads that were prepared from SMAR1 overexpressed and knockdown HCT116 cells. Results showed that HCT116 cells that were overexpressed with SMAR1 exhibited very less number of chromosomal breaks even after exposure to IR (Figure 4a and Supplementary Figure S5). On the contrary, knockdown of SMAR1, in combination with IR, resulted in increased chromosomal aberrations, highlighting the indispensability of SMAR1 for repair upon IR-induced DNA damage. To comprehensively elucidate the role of SMAR1 in DNA damage repair, we performed an *in vivo* end joining assay using pGL2-Luc vector, a microhomologous DNA damage repair reporter system. In this assay, HindIII- or EcoRI-linearized pGL2-Luc vector was transfected in HCT116 cells that were previously transduced with either Ad-SM or sh3 lentivirus. After 48 h of transfection, end joining efficiency was estimated by measuring luciferase expression that will only be achieved once plasmid is precisely rejoined in its circular form. Of note, EcoRI cleaves within the coding region of *luciferase* gene, therefore precise end joining of the short overhangs involves microhomology-directed DNA repair to restore Luc activity. On the contrary, HindIII cuts inside the linker region located between the SV40 promoter and Luc coding sequence, hence precise end joining is dispensable for restoration of Luc activity. Quantification of Luc activity in SMAR1 overexpressed cells that were transfected with EcoRI-linearized pGL2 plasmid DNA showed significant recovery (74%), whereas SMAR1 knockdown counterpart showed only 38% recovery in Luc activity (Figure 4b). However, simple end ligation, as occurs in the case of HindIII-linearized plasmids, is not affected by knockdown of SMAR1. In summary, these results strongly emphasized the crucial role of SMAR1 in the repair of damaged DNA.

Considering that accurate repair of damaged DNA requires the arrest of cell cycle progression,^31^ we investigated the role of SMAR1 in altering cell cycle checkpoints in response to IR-induced DNA damage. Towards this, HCT116 cells that were previously transduced with Ad-SM or sh3 lentivirus were either left untreated or irradiated (10 Gy) and subsequently stained with propidium iodide (PI). Analysis of different phases of cell cycle indicated that SMAR1 positively regulates the onset of IR-induced G2/M checkpoint as evidenced by increased 4N-DNA content in cell population (Figure 4c and Supplementary Figure S6). Strikingly, SMAR1-deficient cells failed to exhibit G2/M checkpoint, and instead showed elevated apoptotic population. We further set out to decipher the molecular mechanism underlying SMAR1-mediated G2/M arrest. Earlier reports attributed Chk2 as a key regulatory checkpoint kinase that is phosphorylated at threonine 68 by ATM in response to IR and regulates G2/M transition.^32^ To check whether SMAR1 regulates IR-induced G2/M arrest by modulating Chk2 phosphorylation, we performed immunoblot analysis in control and SMAR1 overexpressed HCT116 cells. Our results demonstrated that SMAR1 overexpression is associated with enhanced Chk2 phosphorylation upon IR (Figure 4d). Moreover, we observed a concomitant increase in the phosphorylation of downstream G2/M checkpoint regulators, such as Cdc25C and Cdc2. However, we did not observe any change in the phosphorylation of Chk2 upon SMAR1 overexpression in control cells (Figure 4d, lane 3), strongly suggesting that SMAR1 induces G2/M arrest only upon IR-induced stress. Decreased phosphorylation of histone H3 at serine 10, further supported that indeed SMAR1 delays progression of cells from G2 to M phase upon IR. To further ascribe ATM-dependent functionality of SMAR1-induced cell cycle arrest, SMAR1 overexpressed cells were incubated with KU-55933 before IR. Results from these experiments indicated that SMAR1-mediated IR-induced G2/M arrest was reversed upon ATM inhibition (Figure 4d, lane 5), affirming the indispensability of ATM for the efficient functioning of SMAR1 in IR-induced modulation of cell cycle checkpoint. To further correlate the levels of Chk2 phosphorylation with its kinase activity, we performed Chk2 kinase activity assays. Results showed significantly enhanced kinase activity of immunoprecipitated phospho-Chk2 in irradiated HCT116 cells that were overexpressed for SMAR1 (Figure 4e). Together, these data strongly implicate SMAR1 as a key cell cycle regulator, especially during G2/M checkpoint upon DNA damage to ensure efficient repair.

### SMAR1 regulates IR-induced apoptosis and orchestrates Ku70–Bax interaction

**Figure 5:**
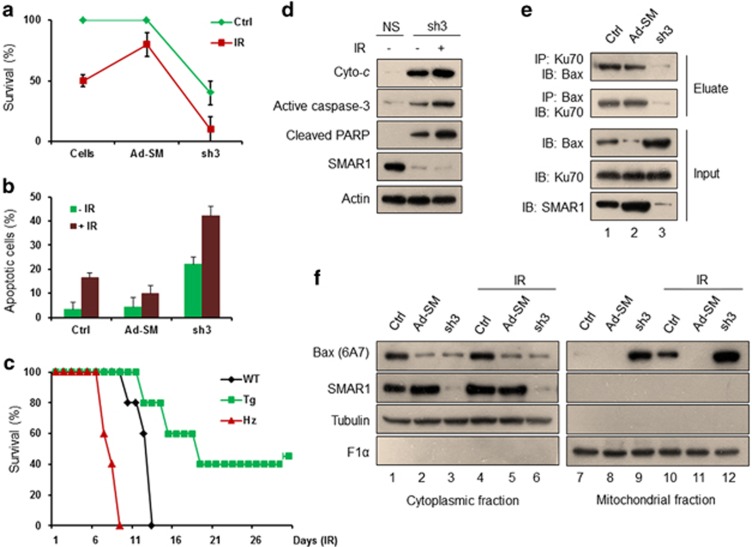
SMAR1 mediates anti-apoptotic response upon IR by regulating Ku70–Bax association. (**a**) Colony formation assay was performed to check the effect of SMAR1 on cell survival. Control, SMAR1 overexpressed (Ad-SM) and knockdown (sh3) HCT116 cells were trypsinized and seeded in 35 mm culture dishes. Eight hours later, cells were irradiated (10 Gy), allowed to grow for 2 weeks, and stained with crystal violet. Percentage survival of control cells was set as 100% to calculate the survival of treated cells. Data shown are representative of three independent experiments and bars represent S.D. (**b**) Statistical representation of percentage apoptotic cells, as determined by apoptosis assay in control and irradiated (10 Gy, 36 h) HCT116 cells upon SMAR1 overexpression and knockdown. Error bars represent S.D. from three independent experimental repeats. (**c**) Effect of SMAR1 on radiosensitivity was analyzed by studying the survival of SMAR1 transgenic (Tg), SMAR1 wild type (WT) and SMAR heterozygous knockout (Hz) mice in response to whole-body irradiation (7 Gy). (**d**) Western blot analysis of indicated apoptotic marker proteins in HCT116 cells transduced with either NS or sh3 lentivirus upon IR (10 Gy, 12 h). (**e**) IP assays to check the effect of SMAR1 on Ku70–Bax association. Lysates from control and cells transduced with either Ad-SM or sh3 were immunoprecipitated with either Ku70 or Bax and the eluates were further immunoblotted with indicated antibodies. (**f**) Western blot analysis of active Bax (6A7), SMAR1 and indicated control proteins in the cytoplasmic (left panel) and mitochondrial (right panel) fractions of control and Ad-SM or sh3 lentivirus-transduced HCT116 cells that were either left untreated or irradiated (10 Gy, 12 h)

Critical analysis of our cell cycle data indicated enhanced apoptosis in irradiated cells that were knockdown for SMAR1. To further substantiate IR-induced apoptosis upon SMAR1 knockdown, we performed colony formation assays in control or irradiated SMAR1 overexpressed and knockdown HCT116 cells. Examination of colony formation by crystal violet staining revealed that SMAR1 overexpressed cells exhibited increased radioresistance, whereas SMAR1 knockdown cells showed enhanced apoptosis (Figure 5a). Next, we assayed cellular apoptosis using MitoCapture apoptotic detection kit (Calbiochem, San Diego, CA, USA) and quantification of apoptotic populations revealed that SMAR1-deficient cells are highly sensitive to IR, as evidenced by 46.05% apoptotic population (Figure 5b and Supplementary Figure S7). On the other hand, SMAR1 overexpressed cells showed only 10.04% apoptotic population upon IR. Altogether, these results conclusively underscore the significance of SMAR1 in imparting resistance toward IR-induced stress. In accordance with these observations, we performed whole-body irradiation (7 Gy) experiments in SMAR1-overexpressing transgenic, SMAR1 heterozygous knockout and wild-type littermate mice to highlight the crucial role of SMAR1 in survival upon DNA damage. Interestingly, 40% of SMAR1 transgenic mice survived even after 20 days of IR, whereas SMAR1 heterozygous knockout mice died within 9 days of IR (Figure 5c). We further evaluated the expression pattern of apoptotic markers upon SMAR1 knockdown. Results indicated an increase in the expression of cytochrome *c*, active caspase-3 and cleaved PARP upon SMAR1 knockdown and this increase was further manifested upon IR (Figure 5d). Altogether, above results indicated that SMAR1 generates a strong anti-apoptotic response upon genotoxic stress.

Earlier studies have reported that acetylation of Ku70 alters its association with Bax, which translocates to mitochondria and induces cellular apoptosis.^14^ As SMAR1 modulates the acetylation status of Ku70, we investigated for any possible correlation between SMAR1 expression and Ku70–Bax association. IP assays in the HCT116 cells that were either overexpressed or knockdown for SMAR1 expression revealed that SMAR1 knockdown resulted in severely impaired Ku70–Bax interaction (Figure 5e, lane 3). This perturbation in Ku70–Bax association can be plausibly explained by the enhanced acetylation of Ku70 upon SMAR1 knockdown. It is noted, once Bax is free from its association with Ku70, it translocates to mitochondria and initiates apoptosis.^14^ Considering that SMAR1 positively regulates the association of Ku70 with Bax, we further reasoned that SMAR1 modulates the cellular localization of Bax. Towards this, western blot analysis of subcellular fractions revealed impaired mitochondrial translocation and cytoplasmic retention of active Bax (6A7) in SMAR1 overexpressed HCT116 cells (Figure 5f, lanes 5 and 11). In contrast, we observed enrichment of Bax in the mitochondrial fractions of SMAR1 knockdown cells, which further increased upon IR (Figure 5f, lanes 9 and 12). It is worth mentioning here that reduced expression of Bax in the cytoplasmic fraction upon SMAR1 overexpression is attributed to SMAR1-mediated transcriptional repression of Bax.^7^ Overall, our results convincingly demonstrate that SMAR1 regulates cell survival upon DNA damage by modulating the association of Ku70 and Bax.

## Discussion

**Figure 6:**
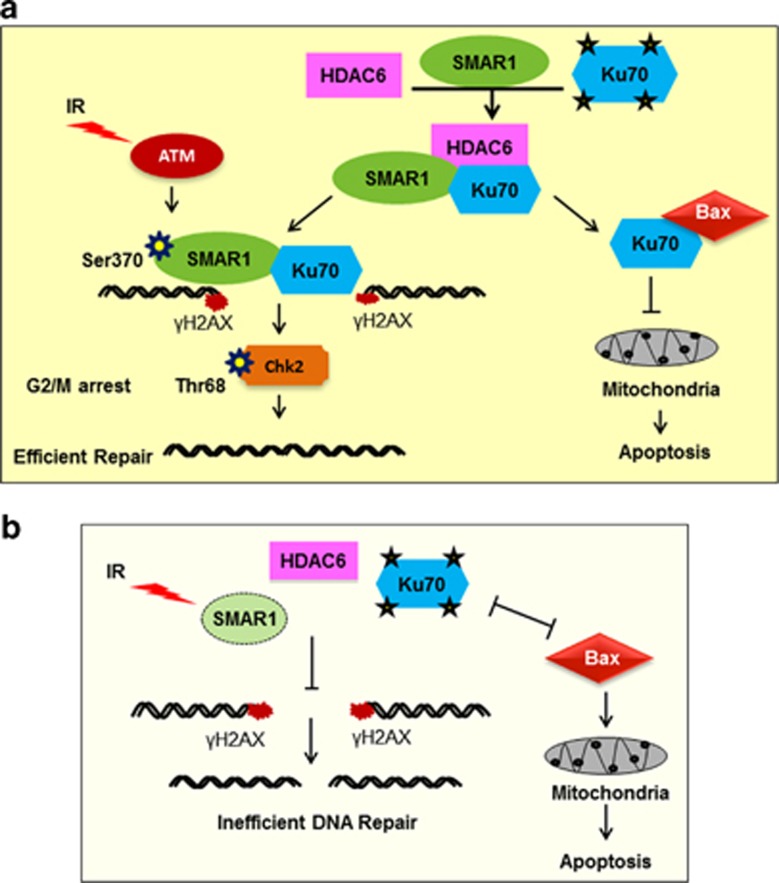
Proposed model for the role of SMAR1 in IR-induced DNA damage repair. (**a**) SMAR1 forms a triple complex with Ku70 and HDAC6, thus maintains Ku70 in a deacetylated state. Upon IR, SMAR1 gets phosphorylated by ATM at Ser370 and favors the recruitment of deacetylated Ku70 to the DSB sites. Moreover, SMAR1 also favors G2/M arrest, thus providing damaged cells ample time for efficient repair. On the other hand, deacetylated Ku70 interacts with Bax and regulates Bax-mediated apoptosis. (**b**) SMAR1 knockdown results in increased acetylation of Ku70 due to perturbation of triple complex between SMAR1, Ku70 and HDAC6. Acetylated Ku70 does not bind to DSB sites, leading to inefficient DNA repair and does not interact with Bax as well, resulting in apoptotic translocation of Bax to mitochondria

Our findings highlight the significance of Ku70–SMAR1–HDAC6 association, which underlines an intricate crosstalk between the various regulators and eventually determines the cell fate. Till date, many proteins have been reported to interact with Ku70 and thereby regulate either DNA damage repair or apoptosis.^20,33,34^ Although various HATs and HDACs have been proposed to have a significant role in the modulation of Ku70 acetylation or deacetylation,^14,18,19^ the involvement of mediator proteins in Ku70 acetylation still remain obscure. Notably, our study for the first time provides evidences of an unidentified mechanism for cell survival that involves SMAR1-mediated fine tune regulation of Ku70 deacetylation through HDAC6, which subsequently results in efficient DNA repair and reduced apoptosis (Figure 6a). A step ahead, SMAR1 was found to be essential for efficient recruitment of Ku70 to damage-associated chromatin fraction to carry out repair, as evidenced by increased chromosomal aberrations and defective DNA repair upon SMAR1 knockdown (Figure 6b). Here, marked reduction in the repair efficiency upon SMAR1 knockdown could be attributed to perturbation of SMAR1–Ku70–HDAC6 triple complex and subsequent increase in Ku70 acetylation, leading to defective binding of acetylated Ku70 to damaged DNA.

One of the earliest events in the aftermath of DNA damage is the ATM-mediated phosphorylation of various factors, which in most cases is followed by distinct foci formation of such phosphorylated proteins.^28^ These foci contain numerous molecules of repair proteins and close proximity of all key players ensures efficient repair. IR-induced activation, ATM-mediated phosphorylation of SMAR1 followed by its relocalization to DSB sites, and colocalization with *γ*H2AX strongly affirms the crucial role of SMAR1 in early response upon DNA damage.

Activation of checkpoint regulators induces arrest in ongoing cell cycle that provides necessary time for repair of damaged DNA before the transmission of wrong genetic information to following generations. In this study, we observed that upon IR-induced DSB generation, SMAR1 favors G2/M arrest via increased phosphorylation of Chk2 at threonine 68. We hypothesize that, being a MARBP, recruitment of SMAR1 near DSB sites might serve as a scaffold for interaction between the components of DDR signaling that favors the induction of checkpoints. Notably, DNA-PK augments the phosphorylation of Chk2 by ATM,^35^ hence increased phosphorylation of Chk2 upon SMAR1 overexpression can be reasoned to enhanced recruitment of Ku70 to DSB sites, which results in the formation of holoenzyme DNA-PK. Furthermore, recruitment of SMAR1, Ku70 and other repair factors near damaged DNA might induce crosstalk between the closely juxtaposed multi-subunit signal transduction complexes, resulting in the amplification of DNA damage signal, and activation of checkpoint regulators and downstream effector proteins such as Cdc25c and Cdc12, which allow sufficient time for a cell to accurately repair damaged DNA.

This study further highlighted that SMAR1 is indispensable for survival upon DNA damage, whereas its deficiency leads to increased apoptosis. SMAR1 has already been shown to repress various pro-apoptotic genes such as Bax and Puma.^7^ However, the alternate mechanism by which SMAR1 manifests its anti-apoptotic function is entirely novel in this study. On one hand, SMAR1 regulates the expression of Bax at transcriptional level by simultaneously inducting p53 deacetylation through HDAC1 to generate anti-apoptotic response. Whereas, SMAR1 also displays posttranslational regulation and functional inhibition of Bax by deacetylation of Ku70 via HDAC6 that subsequently strengthens the Ku70–Bax association, preventing apoptotic translocation of Bax to mitochondria. Here, an interesting question arises that why SMAR1 exhibits an alternate mode of regulation on the function of Bax. One potential answer is that cell needs additional players, which salvage the emergency situation during genotoxic stress by providing efficient cell recovery alternatives. Therefore, it is highly possible that retention factors, such as SMAR1, function in synergistic manner to prevent the relocation of Bax upon DNA damage, even before it is transcriptionally shut down.

In summary, we propose that SMAR1 is a master regulator of cell fate in response to IR-induced DNA damage and our findings highlight the significance of Ku70–SMAR1–HDAC6 association that underlies a complex crosstalk between the various regulators that eventually determine the cell fate. Understanding this rheostat system, which critically balances the cell fate, will be helpful in rationalizing the modes of cell survival, attempting to develop novel and advanced therapeutic tools.

## Materials and Methods

### Cell culture, plasmids and mice

HCT116, HEK 293T, MCF-7 and ATM-MEFs were cultured in DMEM medium (Invitrogen, Darmstadt, Germany) supplemented with 10% fetal bovine serum (FBS, Invitrogen) and 100 U/ml of antibiotic (penicillin–streptomycin; Invitrogen). A-T lymphoblastoid cell lines L3 (ATM deficient) and C3ABR (ATM proficient) were cultured in RPMI medium (Invitrogen). SMAR1 transgenic and wild-type mice were maintained as previously described^36^ and all experiments were performed as per guidelines of animal ethical committee, NCCS. SMAR1 heterozygous knockout mice were generated at Ozgene (Perth, WA, Australia). Exon 2 of SMAR1 was flanked by loxP sites and a phosphoglycerate kinase-neo cassette flanked by FLP recombinase target sites was used for selection. Following homologous recombination of the vector in C57BL/6 embryonic stem cells and establishment of germline transmission, the PGK-neo cassette was excised using the FLP recombinase, leaving exon 2 flanked by loxP sites. As homozygous SMAR1 knockout mice were embryonic lethal, heterozygous knockout mice were used for experimental purposes. Transfection of full-length p3X-Flag-SMAR1, truncations of SMAR1 with p3X-Flag back bone (1–160, 160–350 and 350–548 residues) and serine 370 alanine mutant of SMAR1 (generated by using site-directed mutagenesis kit, Stratagene, La Jolla, CA, USA) were carried out in Opti-MEM medium (Invitrogen) supplemented with 0.5% FBS using Lipofectamine 2000 (Invitrogen). Control and custom synthesized siRNA for SMAR1 (Ambion, San Diego, CA, USA) were used at 300 nM concentration for 24 h. To induce DNA damage, cells were exposed to *γ*-irradiation using gamma chamber with ^137^Cs source as per indicated doses. Whenever needed, ATM inhibitor KU-55933 (10 *μ*M; Tocris Bioscience, Bristol, UK) was added to the media 2 h before IR. HDAC6-specific inhibitor tubacin (5 *μ*M; Sigma, St. Louis, MO, USA) was added to media and incubated for 1 h.

### IP assay and immunoblotting

Cells were harvested and lysed in either whole-cell lysis buffer (50 mM Tris-Cl pH 7.4, 0.5% TritonX-100, 5 mM EDTA, 250 mM NaCl, 50 mM NaF, 0.5 mM Na-Orthovanadate) or CHAPS IP buffer (0.3% CHAPS, 40 mM Hepes pH 7.5, 120 mM NaCl, 1 mM EDTA, 10 mM pyrophosphate, 10 mM glyerophosphate, HALT phosphatase) supplemented with complete protease inhibitor cocktail (Roche, Mannheim, Germany) followed by incubation on ice for 30 min and centrifugation at 12 000 *g* for 20 min at 4 °C. For IP, supernatants were incubated with protein A/G beads (upstate) that were covalently conjugated with control immunoglobulin G or primary antibody. Antibody–bead complex is covalently cross-linked using 10 mg/ml of dimethyl pimelimidate dihydrochloride (DMP; Sigma) for 45 min at room temperature and 5 mg/ml of DMP for 30 min. Unused DMP is eliminated by washing with 50 mM and 200 mM tri-ethanolamine (Sigma). The antibody–bead complex is equilibrated with either IP buffer (0.1% NP-40 in PBS) or CHAPS IP buffer, and incubated with cell lysate at 4 °C for 4 h with rotation. Bound protein is subsequently eluted and subjected to SDS-PAGE and western blotting. The following primary antibodies were used: SMAR1/BANP (Bethyl Laboratories, Montgomery, AL, USA), Ku70, Ku80, active caspase-3, ATM, phospho-ATM Ser1981, cleaved PARP, F1*α*, active Bax (6A7), cytochrome *c*, *α*-tubulin, DNA-PKcs (Santa Cruz, Santa Cruz, CA, USA), Flag, actin, HDAC6 (Sigma), acetyllysine, H3, H4, phospho-Chk2 Thr68, total Chk2, phospho-Cdc25C Ser216, phospho-Cdc12 Tyr15, phospho-H3 Ser10, Bax (Cell Signaling, Frankfurt, Germany) and *γ*H2AX-FITC (upstate).

### Immunofluorescence analysis and laser microirradiation

Cells were processed for immunofluorescence analysis as previously described.^7^ Slides were observed under the fluorescence microscope (Carl Zeiss Axio Imager.Z2, Jena, Germany). For microirradiation studies, cells were subjected to localized damage in the form of specific laser tracts through a UV-A laser beam. Cells were seeded in multi-chamber glass slides (Nunc, Rochester, NY, USA) and pre-sensitized with 10 *μ*M 5-bromo-2′-deoxyuridine (Sigma) in phenol red-free medium (Invitrogen) for 24 h. Laser microirradiation was performed by Axio observer D1 microscope (Carl Zeiss), which is supplemented with PALM microbeam. Cells were exposed to 405 nm laser diode (6 mW) using PALM robo software 4.3 SP1 (Jena, Germany), focused through a × 40 objective to yield a spot size of 0.5–1 mm. The time of exposure was set in fast scanning mode with laser settings in such a range to generate a detectable laser path without causing physical damage to the cells. After microirradiation, imaging of cells was done as described above. Immunostaining for Ku70 was done as described previously.^37^

### *In vitro* kinase assay

*In vitro* kinase assay was performed as described earlier.^38^ Briefly, endogenous ATM was immunoprecipitated for an hour at 4 °C with protein A/G beads from the cleared supernatants of the irradiated (10 Gy) cells that were lysed in modified TGN buffer (50 mM Tris pH 7.5, 50 mM *β*-glycerophosphate, 150 mM NaCl, 1mM dithiothreitol (DTT), 1% Tween-20, 0.3% Nonidet P-40, 1 mM phenylmethylsulfonyl fluoride (PMSF), 1mM sodium fluoride) supplemented with 1 × protease inhibitor cocktail (Roche). After one wash with TGN buffer, the beads were subsequently washed twice in kinase buffer (10 mM HEPES pH 7.5, 50 mM *β*-glycerophosphate, 50 mM NaCl, 10 mM MgCl_2_, 1 mM DTT and 10 mM MnCl_2_). Finally the immunoprecipitates were resuspended in kinase buffer containing 5 *μ*M ATP, 10 *μ*Ci of (*γ*-^32^P) ATP and 1 *μ*g purified GST-fusion substrates, and incubated for 30 min at 30 ºC. The kinase reaction was stopped by adding 2 × SDS loading buffer followed by 12% SDS-PAGE and autoradiography.

### Cellular fractionation

Isolation of chromatin and non-chromatin fractions was done as described previously.^39^ Briefly, cells were suspended in PBS supplemented with 2 mM NaVO_4_, 25 mM NaF, 5 mM PMSF and aprotinin, followed by incubation in buffer A (100 mM NaCl, 300 mM sucrose, 3 mM MgCl_2_, 10 mM PIPES of pH 6.8, 1 mM EGTA, 0.2% Triton X-100, 2 mM NaVO4, 25 mM NaF, 5 mM PMSF and aprotinin) for 30 min at 4 °C and centrifugation. The supernatant (non-chromatin fraction) is collected and the pellet (chromatin fraction) is further extracted in IP0.1 buffer (20 mM HEPES of pH 7.6, 10% glycerol, 25 mM MgCl_2_, 0.1 mM EDTA, 0.2% NP-40, 0.1 M potassium acetate, 1 mM NaVO4 and 50 mM NaF).^40^ Isolation of cytoplasmic and mitochondrial fractions was done from cultured cells by using mitochondria isolation kit (Thermo Scientific, Bonn, Germany) as per the guidelines provided by the manufacturer.

### Cell cycle analysis and apoptosis assays

Cells were trypsinized and fixed with 80% chilled ethanol for 2 h at 4 ºC. After treatment with RNase A (10 *μ*g/ml; Sigma), cells were stained with PI (Sigma) followed by acquisition and analysis by FACS calibur cytometer (BD Biosciences, San Jose, CA, USA) using Cell Quest software (BD Biosciences). Apoptosis assays were performed using MitoCapture apoptosis detection kit (Calbiochem) as per the manufacturer's instructions. Briefly, cells were harvested and stained with MitoCapture dye followed by flow cytometry. Healthy cells, wherein the aggregates of dye in the mitochondria emit red fluorescence, are detected in the PI (FL2) channel, whereas apoptotic cells are detected in the FITC (FL1) channel due to green fluorescence of dye monomers that are dispersed throughout the cell. All assays were carried out in triplicates.

### Chk2 kinase activity assay

Analysis of checkpoint kinase activity was performed using K-LISA checkpoint activity kit (Calbiochem) according to the manufacturer's protocol. Briefly, Chk2 was immunoprecipitated from the lysates of HCT116 cells and immunoprecipitates were incubated with biotinylated peptide substrate in the presence of ATP. Phosphorylated substrate is detected with phosphoserine-specific antibody and kinase activity is determined using colorimetric detection.

### *In vivo* NHEJ assay

*In* vivo NHEJ assay using plasmid substrates was done as described.^41^ Briefly, pGL2 vector was linearized with either *Hin*dIII or *Eco*RI, and the linearized fragment was purified by gel extraction method, estimated and transfected. After 48 h of transfection, cells were harvested and assayed for luciferase activity.

### Metaphase spread analysis

Metaphase spreads were prepared as previously described.^42^ Briefly, cells were arrested at metaphase by adding 100 ng/ml of colcemid (Sigma) to the culture media, followed by incubation with pre-warmed hypotonic solution and fixation (methanol:acetic acid in 3 : 1). Finally, 20–50 *μ*l of cell suspension was dropped on a humidified glass slide. Slides were subsequently stained with Giemsa stain (Sigma) and metaphase spreads were observed under bright field microscope (Carl Zeiss Axiovert 200 M) using × 100 objective.

### Protein–protein docking analysis

To perform protein–protein docking of HDAC6, SMAR1 and Ku70, the structural coordinates of HDAC6 (3C5K)^43^ and Ku70 (1JEQ)^10^ were obtained from PDB database. Crystal structure of free domain of Ku70, which is not bound to Ku80, was extracted from the Ku70:Ku80 complex. Next, the structure of SMAR1 was built with I-TASSER online server (Michigan, MI, USA).^44^ The structural coordinates of all the three systems were optimized in the protein preparation wizard of Maestro.^45^ All the missing hydrogen atoms were added and further subjected to energy minimization with OPLS-2005. The interaction models for SMAR1, HDAC6 and Ku70 were obtained with ZDOCK (v.3.0.2; University of Massachusetts, Worchester, MA, USA), which predicts the interacting models by the fast Fourier transform and allows 3D searches of spatial degrees of freedom between the macromolecules.^46^ The predicted docked models of HDAC6–SMAR1–Ku70 were further analyzed with the PyMol (Schrodinger).^47^

## Abbreviations

NM: nuclear matrix
SMAR1: scaffold/matrix-associated region-binding protein 1
DSB: DNA double-strand break
MARBPs: matrix attachment region-binding proteins
IR: ionizing radiation
DNA-PKcs: DNA-dependent protein kinase catalytic subunit
NHEJ: non-homologous end joining
IP: immunoprecipitation
EtBr: ethidium bromide
MEFs: mouse embryonic fibroblasts
PI: propidium iodide
