# Supplementary material for: SMAR1 coordinates HDAC6-induced deacetylation of Ku70 and dictates cell fate upon irradiation
Source: Cell Death Dis. 2014 Oct 9;5(10):e1447–. doi: 10.1038/cddis.2014.397 (PMC4237237; doi:10.1038/cddis.2014.397)
Supplement: Supplementary Figure Legends [file cddis2014397x1.doc]

**Supplementary Figure Legends**

**Figure S1.** (a) HCT116 cells were exposed to different doses of ionizing irradiation (IR) and subjected to immunoblot analysis of SMAR1. Actin was used as a loading control. (b) Graph depicts the densitometry quantification of time-dependent expression of SMAR1 in HCT116 cells upon ionizing irradiation (10 Gy) using QuantityOne software (VersaDoc imaging system, BioRad). Control cell sample (as the value of 1) was used to normalize values of treated samples. Data represents single experiment from three independent experimental repeats. (c) Densitometry quantification of SMAR1 expression in chromatin- and non-chromatin fractions of HCT116 cells that were either left untreated or irradiated (10 Gy, 2 h). (d) Graph depicts the IP assay to check interaction between SMAR1 and Ku70 in control and irradiated (10 Gy, 8 h) HCT116 cells. The data is from a single experiment that is representative of three independent experiments.

**Figure S2.** (a) IP assays to check the *in vivo* association of SMAR1 with Ku70. Control and irradiated (10 Gy, 8 h) HCT116 cell lysates were immunoprecipitated with Ku70 or control IgG followed by immunoblotting the eluates with SMAR1 and Ku70, as shown. 20% fraction of whole cell extract served as an input control. (b-c) IP assays to study SMAR1 and Ku70 interactions in HEK293 (b) and MCF-7 (c) cells. Control and irradiated (10 Gy, 8 h) cell lysates (1mg) were immunoprecipitated with SMAR1 and eluates were further probed for Ku70. (d) GST-pull down assay using recombinant GST and GST-SMAR1 (GS) protein. Thymic extracts (500 µg) from control and irradiated BALB/c mice were incubated with bead-bound protein followed by immunoblotting with Ku70. (e) IP assay to study SMAR1-Ku70 association upon IR (10 Gy) in HCT116 cells at indicated time points. (f) Irradiated (10 Gy) HCT116 lysates were treated with either EtBr (50 μg/ml, 30 min) at 4°C or DNase I (100 U/ml, 20 min) at 37°C. Whole cell lysates were immunoprecipitated with Ku70 and then immunoblotted for DNA-PKcs and Ku70.

**Figure S3.** (a) Western blot analysis of SMAR1 in control and SMAR1-siRNA transfected HCT116 cells. Actin was used as a loading control. (b) Colocalization study of γH2AX (green) and Ku70 (red) in control and SMAR1-siRNA transfected HCT116 cells upon laser microirradiation (1h).

**Figure S4.** (a) Colocalization study of γH2AX (green) and SMAR1 (red) in HCT116 cells upon laser microirradiation (1h). Nuclei were stained with DAPI (blue). (b) IP assay to check the *in vivo* association between γH2AX and SMAR1 in irradiated (10 Gy, 2 h) HCT116 cells. Irradiated cell lysates were immunoprecipitated with γH2AX and the eluates were immunoblotted with the indicated antibodies. (c) Immunoblot analysis of SMAR1 phosphorylation in HCT116 cells with indicated doses of IR using anti-phospho-SMAR1 antibody. (d) IP assay to check the phosphorylation of SMAR1 in Flag-SMAR1 (F-SM) and Flag-S370A mutant (F-Mut) transfected HCT116 cells that were either left untreated or irradiated (10 Gy, 2 h). Cell lysates were immunoprecipitated with Flag antibody and eluates were immunoblotted with phospho-SMAR1antibody (p-SMAR1). (e) Western blot analysis of chromatin-bound fraction for the expression of SMAR1 in HCT116 cells that were either left untreated or treated with caffeine (5 mM), before 1h of irradiation.

**Figure S5.** Quantification of metaphase spreads with chromosomal aberrations from SMAR1 overexpressed (Ad-SM) and knockdown (sh3) HCT116 cells upon IR (10 Gy). The data is representative of ˃50 images (n˃50), which were acquired in various fields from three independent experiments. Error bars represent standard deviation (SD).

**Figure S6.** Propidium iodide staining to study the effect of SMAR1 on IR-induced cell-cycle progression. Different cell-cycle phases were checked in control and SMAR1 overexpressed (Ad-SM) cells or knockdown (sh3) HCT116 cells upon irradiation (10 Gy, 48 h). Cell-cycle analysis of control HCT116 cells and cells transduced with SMAR1-overexpressing adenovirus (Ad-SM) or SMAR1-ShRNA lentivirus (Sh3). Forty eight hours post-transduction, cells were either left untreated or irradiated (10 Gy, 48 h) and thereafter processed for propidium iodide staining.

**Figure S7.** Quantification of apoptotic cell population by apoptotic assay in control HCT116 cells (Ctrl), cells overexpressed (Ad-SM) and knockdown (sh3) for SMAR1 expression upon irradiation (10 Gy, 36 h).

**Table S1.** Analysis of energy content of SMAR1, SMAR1+Ku70 (Dimer), SMAR1+HDAC6 (Dimer) and SMAR1+Ku70+HDAC6 (Trimer).
