# Supplementary figures and images for: SMAR1 coordinates HDAC6-induced deacetylation of Ku70 and dictates cell fate upon irradiation
Source: Cell Death Dis. 2014 Oct 9;5(10):e1447–. doi: 10.1038/cddis.2014.397 (PMC4237237; doi:10.1038/cddis.2014.397)

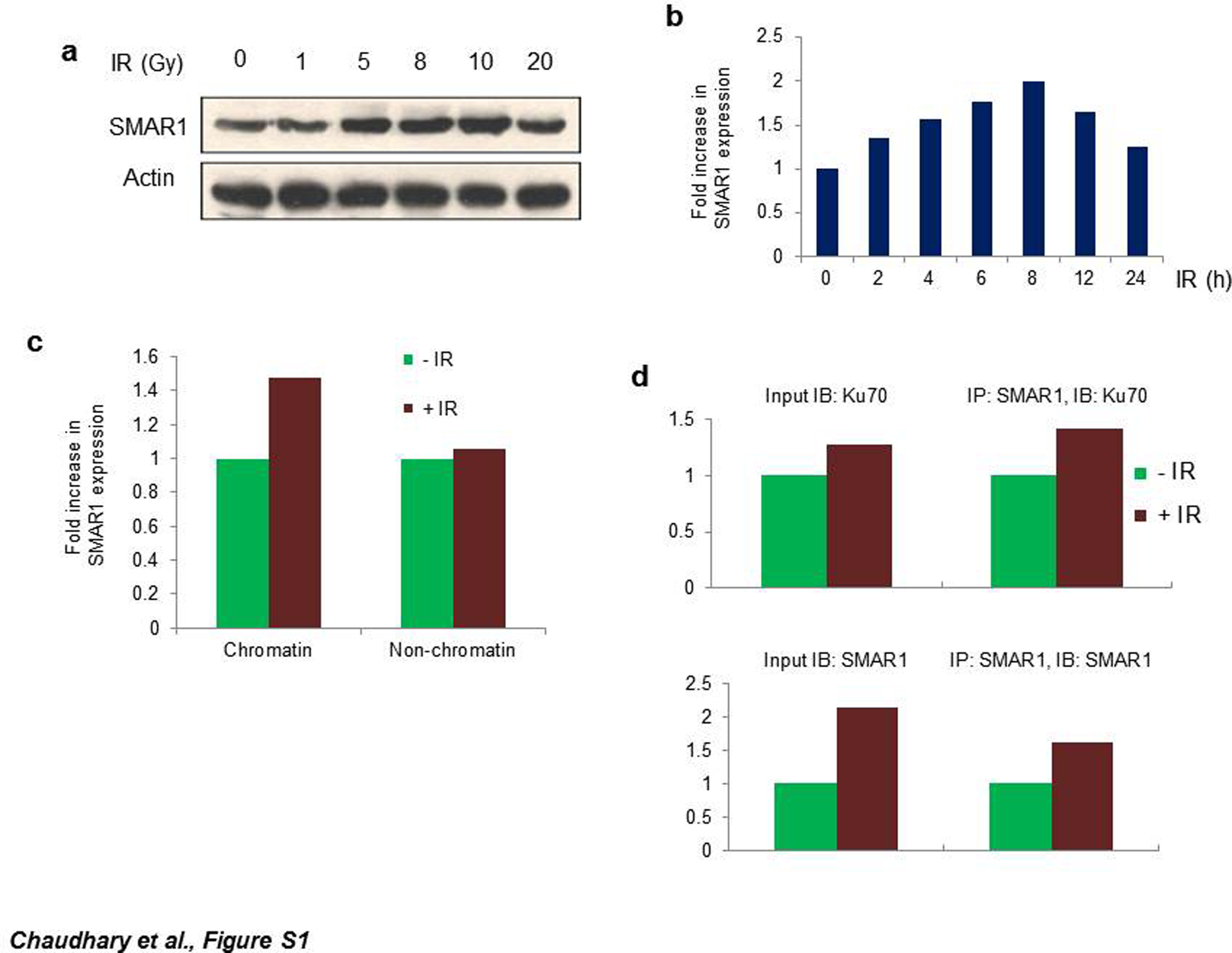

Supplement: Supplementary Figure S1 [file cddis2014397x2.tif]

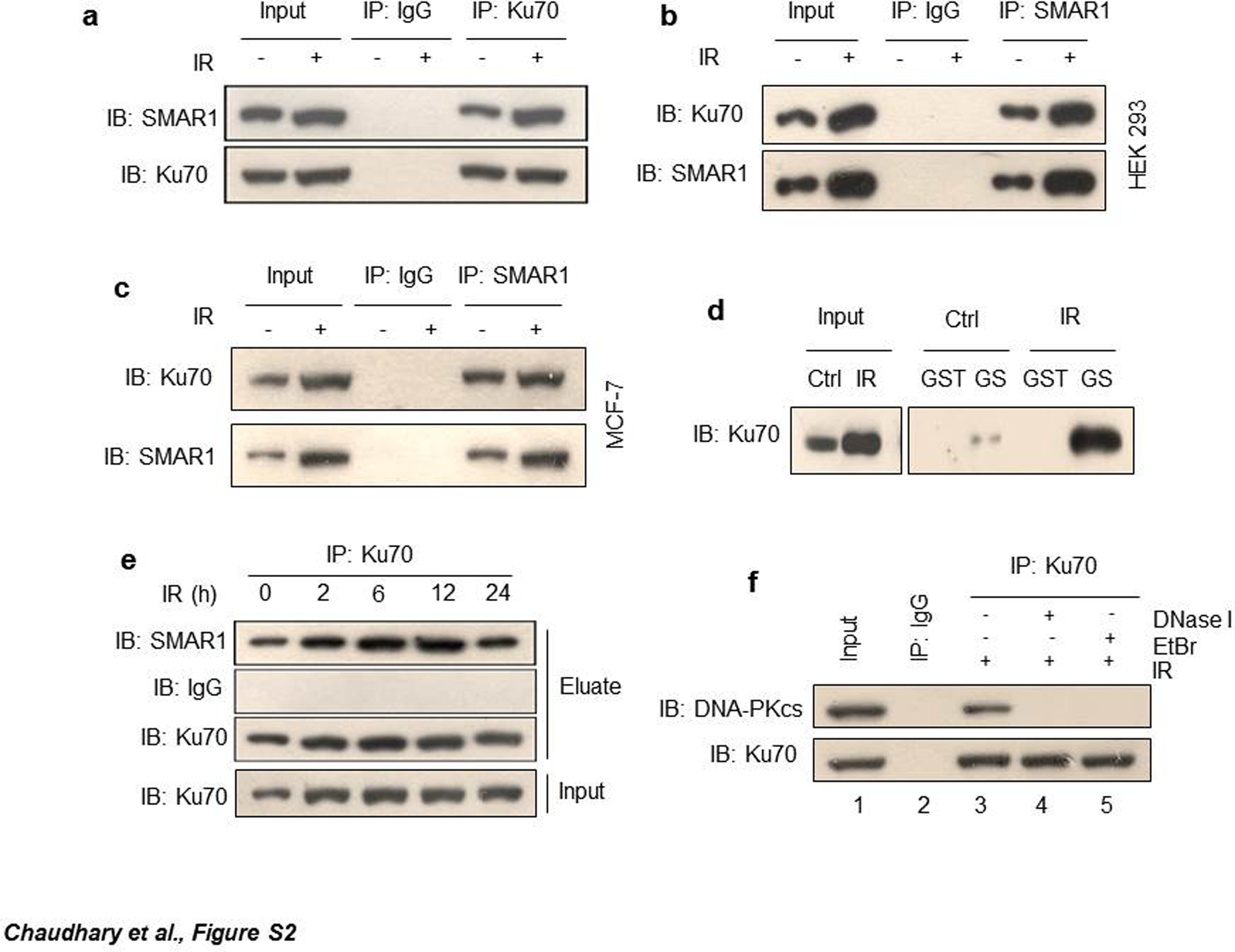

Supplement: Supplementary Figure S2 [file cddis2014397x3.tif]

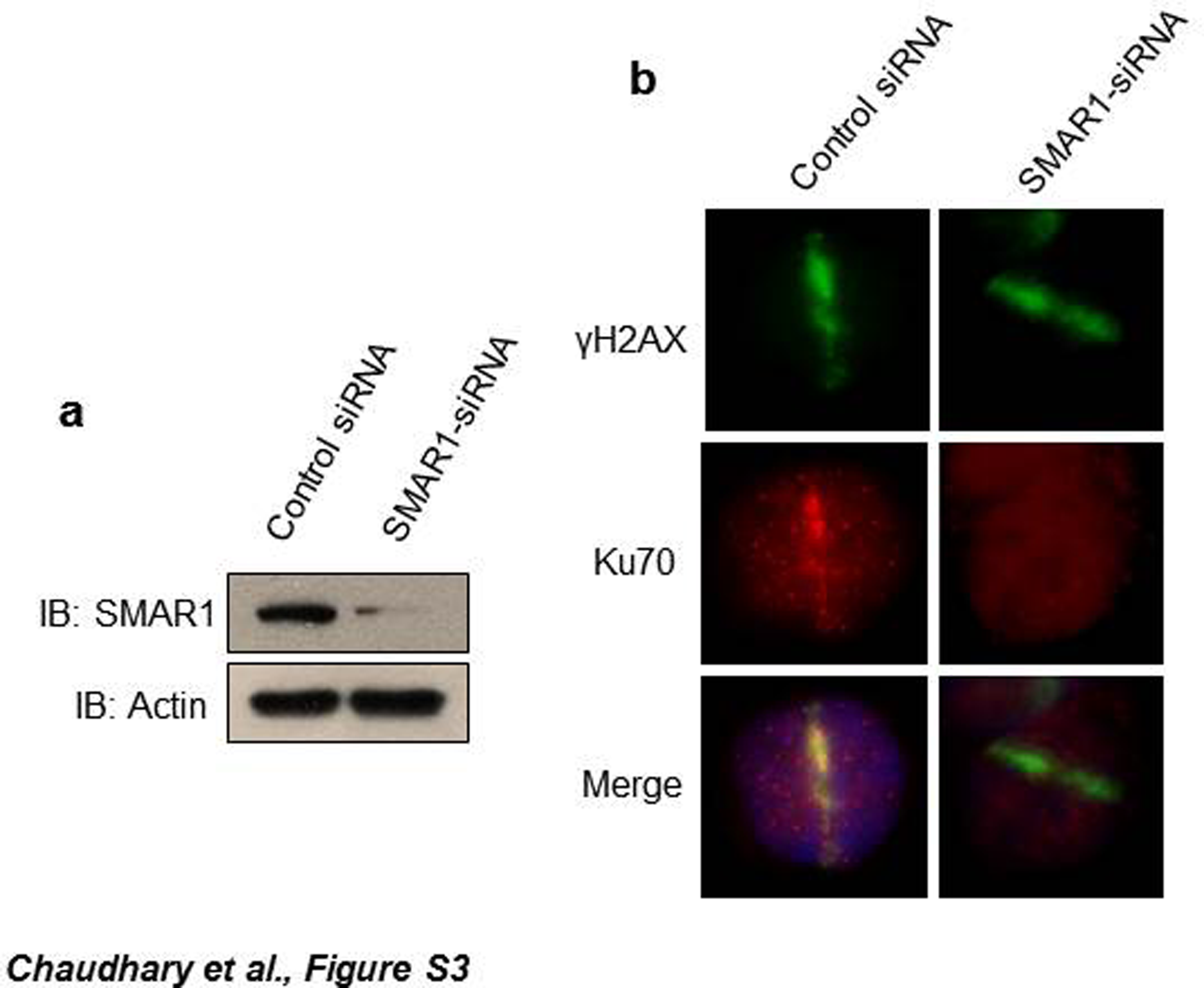

Supplement: Supplementary Figure S3 [file cddis2014397x4.tif]

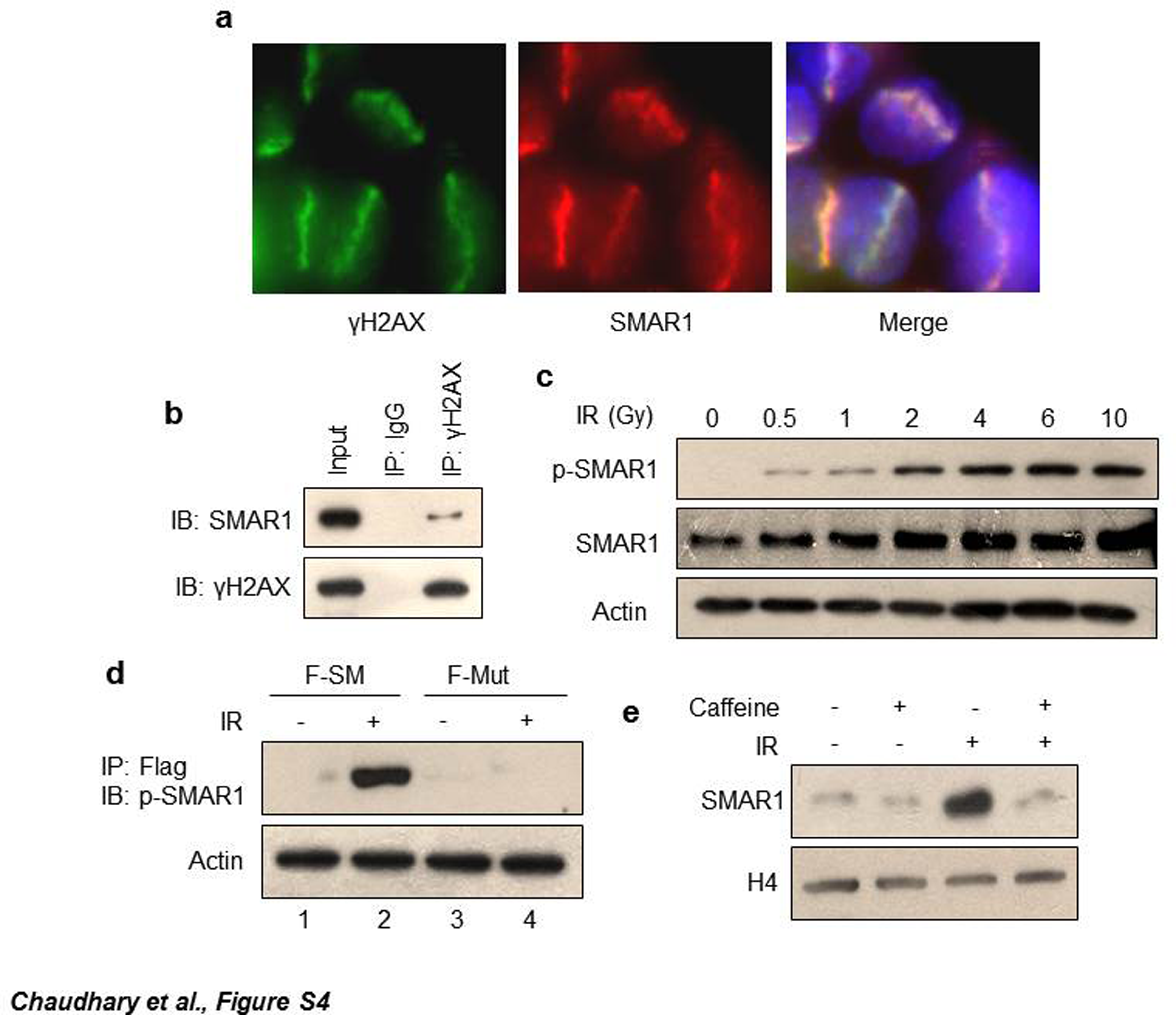

Supplement: Supplementary Figure S4 [file cddis2014397x5.tif]

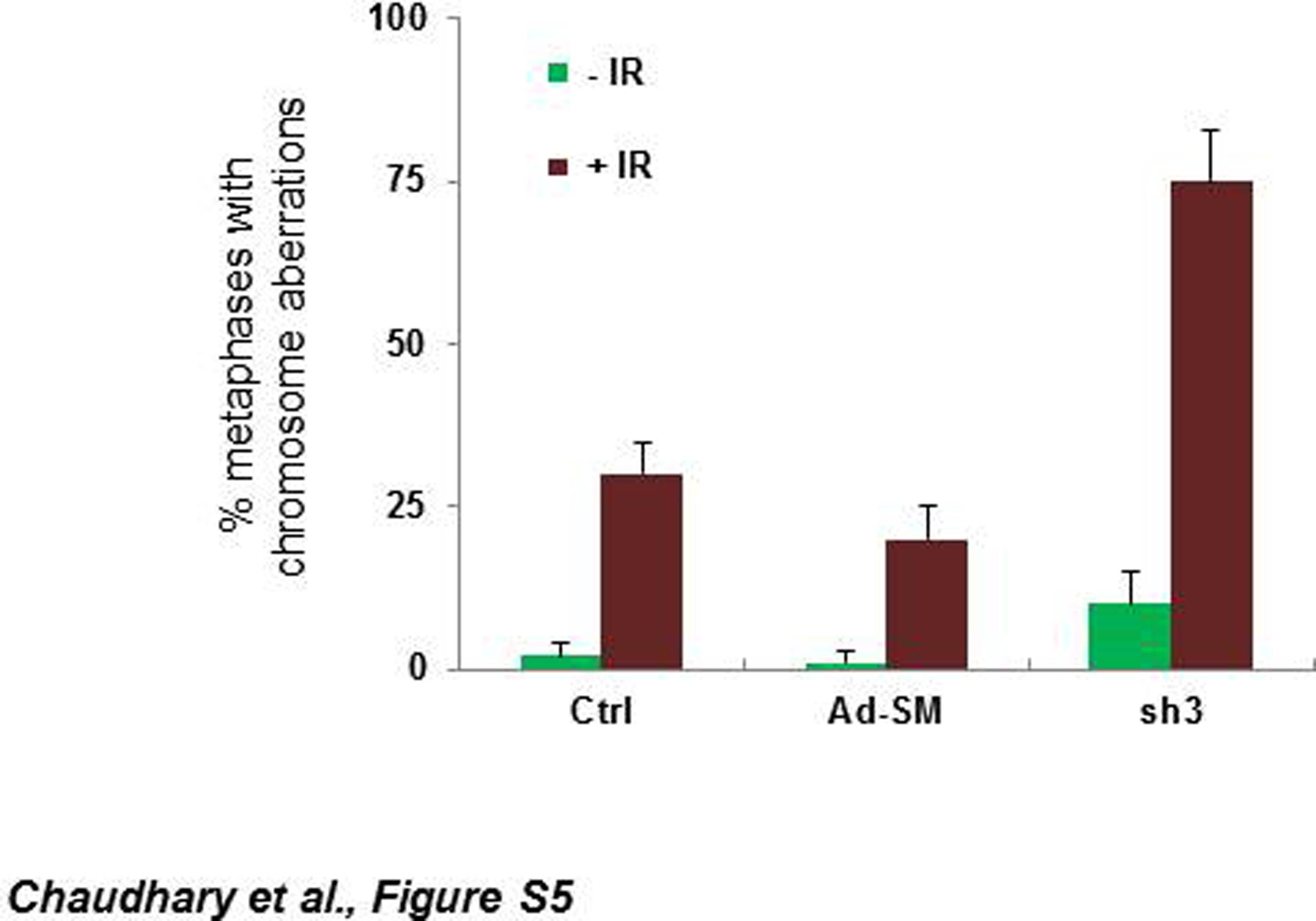

Supplement: Supplementary Figure S5 [file cddis2014397x6.tif]

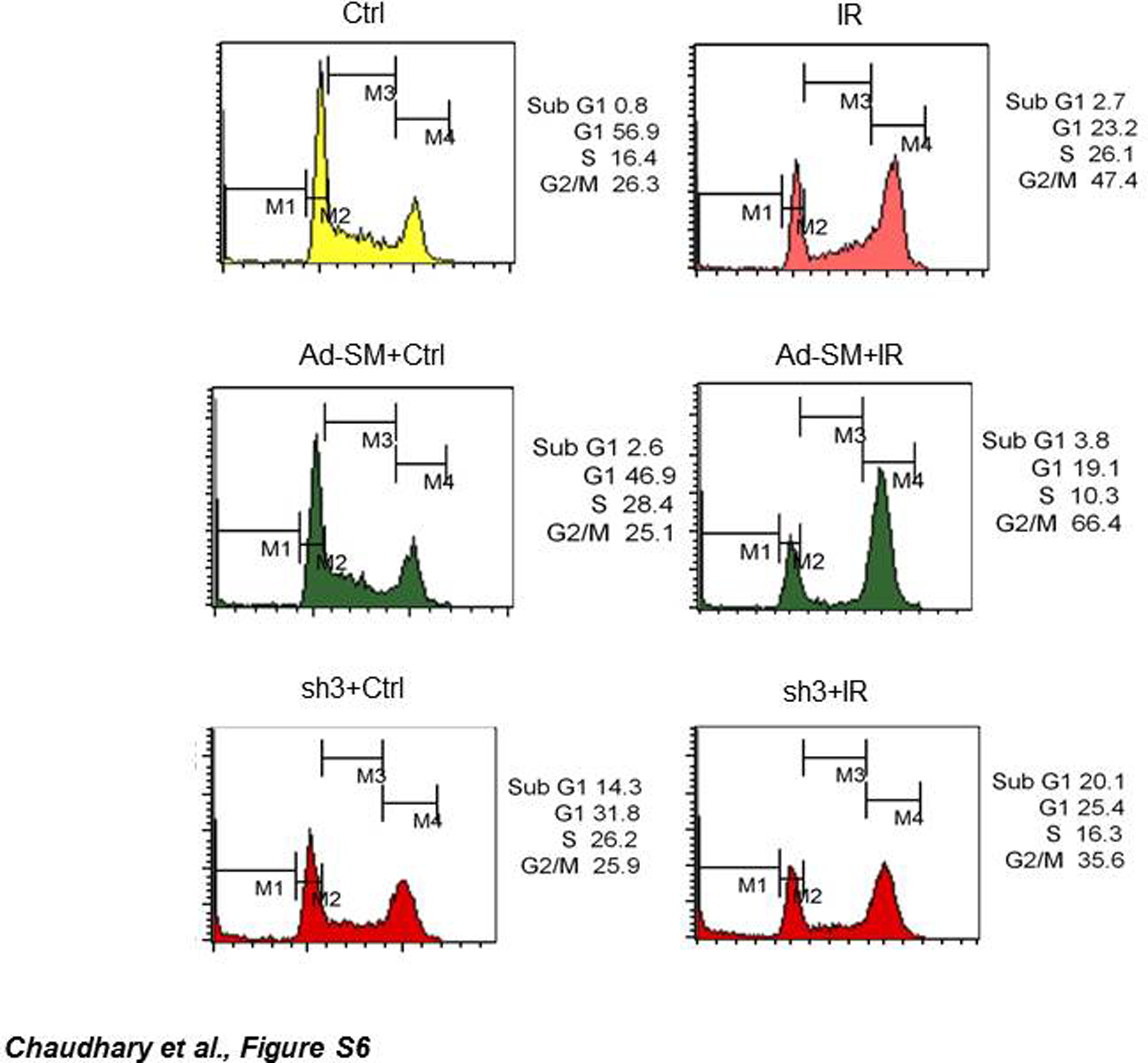

Supplement: Supplementary Figure S6 [file cddis2014397x7.tif]

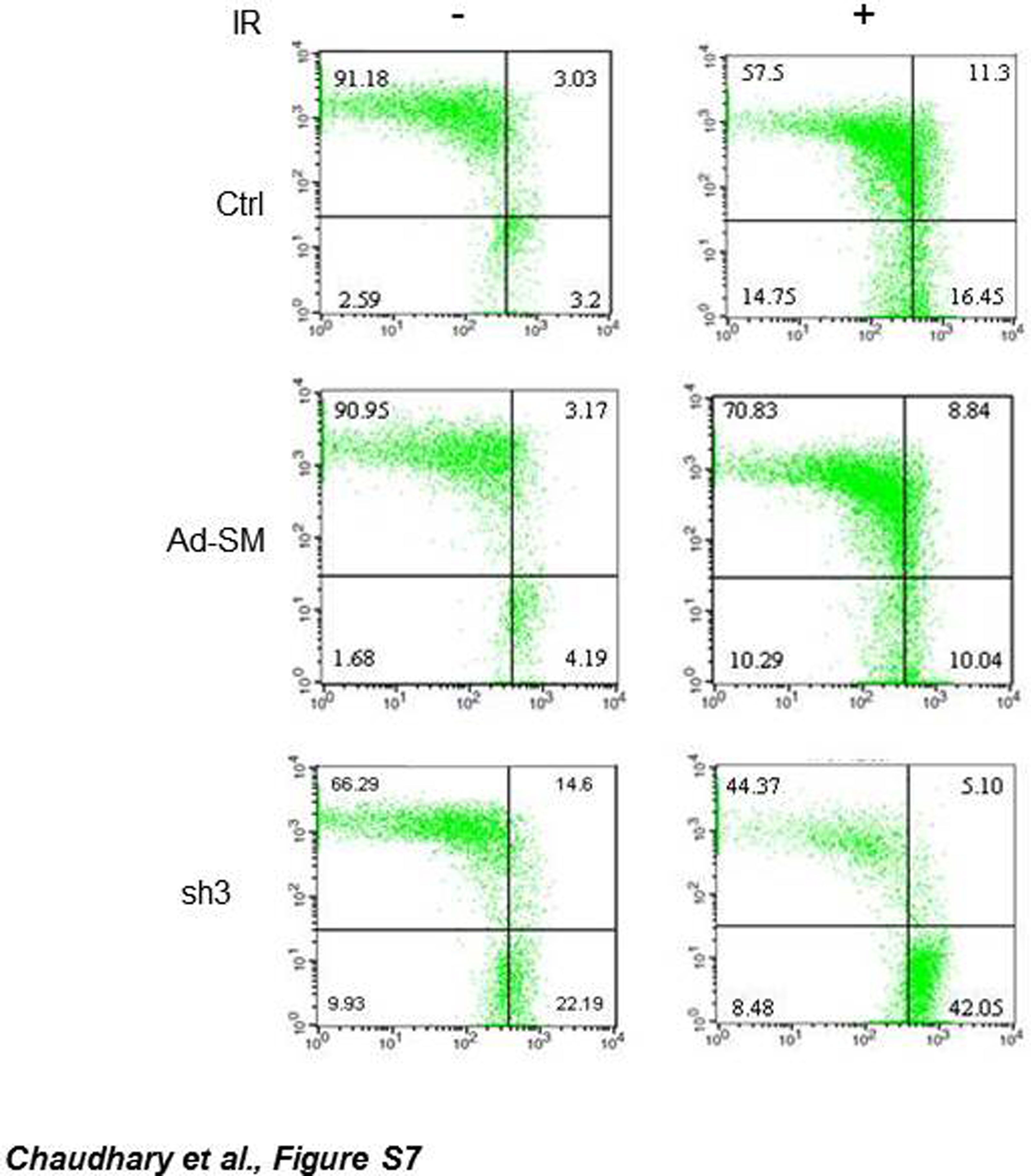

Supplement: Supplementary Figure S7 [file cddis2014397x8.tif]

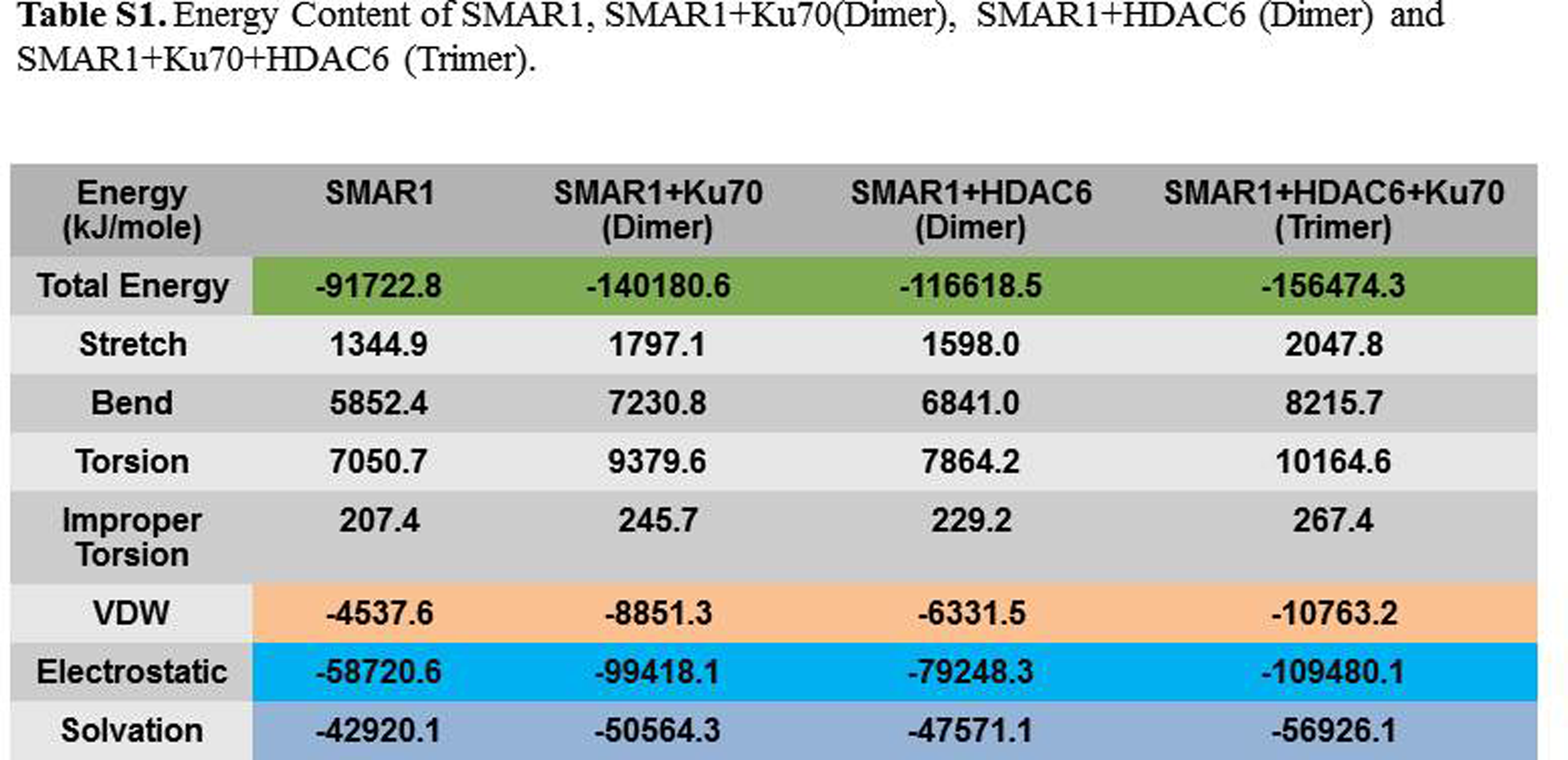

Supplement: Supplementary Table S1 [file cddis2014397x9.tif]
